# Supplementary material for: Risk factors of furazolidone-associated fever
Source: PLoS One. 2022 Apr 8;17(4):e0266763. doi: 10.1371/journal.pone.0266763 (PMC8993017; doi:10.1371/journal.pone.0266763)
Supplement: Attachment — Submitted filename: pdf.pdf [file pone.0266763.s001.pdf]

PLOS ONE

Risk Factors of Furazolidone-Associated Fever

--Manuscript Draft--

**Manuscript Number:**	PONE-D-21-28795
**Article Type:**	Research Article
**Full Title:**	Risk Factors of Furazolidone-Associated Fever
**Short Title:**	Furazolidone-Associated Fever
**Corresponding Author:**	Haibin Dai<br>Zhejiang University School of Medicine Second Affiliated Hospital<br>Hangzhou, Zhejiang CHINA
**Keywords:**	furazolidone; Helicobacter pylori; drug fever
**Abstract:**	Background<br>Furazolidone is a synthetic nitrofuran with a broad spectrum of antimicrobial action and has been widely used in the treatment of Helicobacter pylori (H. pylori) infection. However, its safety remains inconclusive; the drug fever it can cause is frequently misdiagnosed. The aim of this study was to explore the risk factors of furazolidone-associated fever to increase awareness and stimulate further research on this topic.<br>Methods<br>This was a retrospective cohort study of patients referred to a specialist clinic for furazolidone-containing quadruple regimens for H. pylori infection at a tertiary care hospital located in Eastern China between July 2018 and September 2018. We evaluated adult patients who received furazolidone treatment for Helicobacter pylori infection. The exclusion criteria were as follows: (1) patients were pregnant or breastfeeding; (2) patients received furazolidone treatment not for Helicobacter pylori infection; (3) patients had taken antibiotics or any acid suppressant or non-steroidal anti-inflammatory drug in the last 4 weeks; (4) patients had chronic hepatic, renal, or pulmonary disease. Pertinent information was retrieved from medical records and telephone follow-up. All statistical analysis was performed in SPSS version 22.0.<br>Results<br>A total of 1499 patients received polymyxin and met the overall inclusion criterion. Of these 1499 patients, 27 (1.80%) developed drug fever. The mean time between initiation of furazolidone and the onset of fever is 11.00 ± 1.84 days, and the median peak fever was 38.87 ± 0.57 °C. We found no differences in age and past drug allergy between the non-fever and fever groups. Through multiple logistic regression analysis, we found two variables as independent risk factors for furazolidone-associated fever, including gender (OR, 3.16; 95% CI, 1.26–7.91; P =0.014) and clarithromycin (OR, 4.83; 95% CI, 2.17–10.79; P <0.001).<br>Conclusions<br>This retrospective cohort study identified two risk factors for furazolidone-associated fever, which were female and clarithromycin. We also analyzed the characteristics of drug fever during anti- Helicobacter pylori therapy. However, the underlying mechanisms are uncertain and require further research.
**Order of Authors:**	Jiali Zhang
	Chunling Rong
	Chenyang Yan
	Jie Chen
	Wenjun Yang
	Lingyan Yu
	Haibin Dai
**Additional Information:**	
**Question**	**Response**
**Financial Disclosure**	Yes, this project was supported by a grant from the Natural Science Foundation of

Enter a financial disclosure statement that describes the sources of funding for the work included in this submission. Review the submission guidelines for detailed requirements. View published research articles from *PLOS ONE* for specific examples.<br><br>This statement is required for submission and **will appear in the published article** if the submission is accepted. Please make sure it is accurate.<br><br>**Unfunded studies**<br>Enter: *The author(s) received no specific funding for this work.*<br><br>**Funded studies**<br>Enter a statement with the following details:<br>• Initials of the authors who received each award<br>• Grant numbers awarded to each author<br>• The full name of each funder<br>• URL of each funder website<br>• Did the sponsors or funders play any role in the study design, data collection and analysis, decision to publish, or preparation of the manuscript?<br>• **NO** - Include this sentence at the end of your statement: *The funders had no role in study design, data collection and analysis, decision to publish, or preparation of the manuscript.*<br>• **YES** - Specify the role(s) played.<br><br>* typeset	Zhejiang Province (Grant No. LYY19H310013) which was received by JL Zhang, the Foundation from the Health Bureau of Zhejiang Province (Grant No. 2019KY406) which was received by JL Zhang, and the foundation from Zhejiang Pharmaceutical Association (Grant No. 2019ZYY18) which was received by LY Yu.
**Competing Interests**<br><br>Use the instructions below to enter a competing interest statement for this submission. On behalf of all authors, disclose any competing interests that could be perceived to bias this work—acknowledging all financial support and any other relevant financial or non-financial competing interests.<br><br>This statement is required for submission and **will appear in the published article** if	The authors have declared that no competing interests exist.

the submission is accepted. Please make sure it is accurate and that any funding sources listed in your Funding Information later in the submission form are also declared in your Financial Disclosure statement.<br><br>View published research articles from *PLOS ONE* for specific examples.<br><br>**NO authors have competing interests**<br><br>Enter: *The authors have declared that no competing interests exist.*<br><br>**Authors with competing interests**<br><br>Enter competing interest details beginning with this statement:<br><br>*I have read the journal's policy and the authors of this manuscript have the following competing interests: [insert competing interests here]*<br><br>* typeset	
**Ethics Statement**<br><br>Enter an ethics statement for this submission. This statement is required if the study involved:<br><br>• Human participants<br>• Human specimens or tissue<br>• Vertebrate animals or cephalopods<br>• Vertebrate embryos or tissues<br>• Field research<br><br>Write "N/A" if the submission does not require an ethics statement.<br><br>General guidance is provided below. Consult the submission guidelines for detailed instructions. **Make sure that all information entered here is included in the Methods section of the manuscript.**	This study was approved by the medical ethics committee of the Second Affiliated Hospital, Zhejiang University School of Medicine, China (No. 2020-283). The need for informed consent was waived due to the retrospective design of this study. All data collected was kept confidential.

Powered by Editorial Manager® and ProduXion Manager® from Aries Systems Corporation

**Format for specific study types**<br><br>**Human Subject Research (involving human participants and/or tissue)**<br>• Give the name of the institutional review board or ethics committee that approved the study<br>• Include the approval number and/or a statement indicating approval of this research<br>• Indicate the form of consent obtained (written/oral) or the reason that consent was not obtained (e.g. the data were analyzed anonymously)<br><br>**Animal Research (involving vertebrate animals, embryos or tissues)**<br>• Provide the name of the Institutional Animal Care and Use Committee (IACUC) or other relevant ethics board that reviewed the study protocol, and indicate whether they approved this research or granted a formal waiver of ethical approval<br>• Include an approval number if one was obtained<br>• If the study involved *non-human primates*, add *additional details* about animal welfare and steps taken to ameliorate suffering<br>• If anesthesia, euthanasia, or any kind of animal sacrifice is part of the study, include briefly which substances and/or methods were applied<br><br>**Field Research**<br><br>Include the following details if this study involves the collection of plant, animal, or other materials from a natural setting:<br>• Field permit number<br>• Name of the institution or relevant body that granted permission	
**Data Availability**<br><br>Authors are required to make all data underlying the findings described fully available, without restriction, and from the time of publication. PLOS allows rare exceptions to address legal and ethical concerns. See the PLOS Data Policy and FAQ for detailed information.	No - some restrictions will apply

Powered by Editorial Manager® and ProduXion Manager® from Aries Systems Corporation

A Data Availability Statement describing where the data can be found is required at submission. Your answers to this question constitute the Data Availability Statement and **will be published in the article**, if accepted.<br><br>**Important:** Stating 'data available on request from the author' is not sufficient. If your data are only available upon request, select 'No' for the first question and explain your exceptional situation in the text box.<br><br>Do the authors confirm that all data underlying the findings described in their manuscript are fully available without restriction?	
**Describe where the data may be found in full sentences. If you are copying our sample text, replace any instances of XXX with the appropriate details.**<br><br>• If the data are **held or will be held in a public repository**, include URLs, accession numbers or DOIs. If this information will only be available after acceptance, indicate this by ticking the box below. For example: *All XXX files are available from the XXX database (accession number(s) XXX, XXX.).*<br>• If the data are all contained **within the manuscript and/or Supporting Information files**, enter the following: *All relevant data are within the manuscript and its Supporting Information files.*<br>• If neither of these applies but you are able to provide **details of access elsewhere**, with or without limitations, please do so. For example:<br><br>*Data cannot be shared publicly because of [XXX]. Data are available from the XXX Institutional Data Access / Ethics Committee (contact via XXX) for researchers who meet the criteria for access to confidential data.*<br><br>*The data underlying the results presented in the study are available from (include the name of the third party*	The data underlying the results presented in the study are available from Jiali Zhang and the contact information is zjlsally@zju.edu.cn.

and contact information or URL).<br>• This text is appropriate if the data are owned by a third party and authors do not have permission to share the data.<br><br>* typeset	
Additional data availability information:	Tick here if your circumstances are not covered by the questions above and you need the journal’s help to make your data available.

Manuscript Click here to access/download;Manuscript;manuscript.docx

Risk Factors of Furazolidone-Associated Fever

Jiali Zhang[1], Chunling Rong[1], Chenyang Yan[2], Jie Chen[1], Wenjun Yang[1], Lingyan Yu[1], Haibin Dai[1]*

[1]Department of Pharmacy, Second Affiliated Hospital, Zhejiang University School of Medicine, Hangzhou, China

[2] Department of Pharmacy, Marine Police Hospital, Jiaxing, China

*Corresponding author: Haibin Dai, E-mail address: haibindai@zju.edu.cn

Abstract:

Background

Furazolidone is a synthetic nitrofuran with a broad spectrum of antimicrobial action and has been widely used in the treatment of *Helicobacter pylori (H. pylori)* infection. However, its safety remains inconclusive; the drug fever it can cause is frequently misdiagnosed. The aim of this study was to explore the risk factors of furazolidone-associated fever to increase awareness and stimulate further research on this topic.

Methods

This was a retrospective case-control study of patients referred to a specialist clinic for furazolidone-containing quadruple regimens for *H. pylori* infection at a tertiary care hospital located in Eastern China between July 2018 and September 2018. We evaluated adult patients who received furazolidone treatment for *Helicobacter pylori* infection. The exclusion criteria were as follows: (1) patients were pregnant or breastfeeding; (2) patients received furazolidone treatment not for *Helicobacter pylori* infection; (3) patients had taken antibiotics or any acid suppressant or non-steroidal anti-inflammatory drug in the last 4 weeks; (4) patients had chronic hepatic, renal, or pulmonary disease. Pertinent information was retrieved from medical records and telephone follow-up. All statistical analysis was performed in SPSS version 22.0.

Results

A total of 1499 patients received polymyxin and met the overall inclusion criterion. Of these 1499 patients, 27 (1.80%) developed drug fever. The mean time between initiation of furazolidone and the onset of fever is 11.00 ± 1.84 days, and the median peak fever

was 38.87 ± 0.57 ℃. We found no differences in age and past drug allergy between the non-fever and fever groups. Through multiple logistic regression analysis, we found two variables as independent risk factors for furazolidone-associated fever, including gender (OR, 3.16; 95% CI, 1.26–7.91; P=0.014) and clarithromycin (OR, 4.83; 95% CI, 2.17–10.79; P<0.001).

Conclusions

This retrospective cohort study identified two risk factors for furazolidone-associated fever, which were female and clarithromycin. We also analyzed the characteristics of drug fever during anti-*Helicobacter pylori* therapy. However, the underlying mechanisms are uncertain and require further research.

1 Introduction

Helicobacter pylori (H. pylori) infection is common worldwide, with a prevalence from 18.9% to 79.1% in adults [1]. The most commonly recommended therapy worldwide consists of a proton pump inhibitor (PPI), clarithromycin, amoxicillin, and/or metronidazole [2]. Many studies have validated that antibiotic resistance is the clear leading cause of treatment failure [3]. Recently, a review published by Hu et al. revealed that the resistance rates to clarithromycin, metronidazole, and levofloxacin in Chinese patients were 28.9%, 63.8%, and 28.0% [4]. However, the resistance rates of other antibiotics, such as tetracycline and furazolidone, are still low. Studies have shown that the *H. pylori* eradication rates of furazolidone-containing therapies were higher than 80% [5–7]. Because of its high eradication rates and low cost, the Fifth Chinese

National Consensus Report recommended that furazolidone should be used for *H. pylori* eradication treatment [8]. Furazolidone is chemically related to nitrofurantoin, which has primarily been used in humans for the treatment of diarrheal diseases [9]. The most common adverse events of furazolidone including gastrointestinal reactions such as nausea, vomiting, abdominal pain, headache, and allergic reactions were reported [10,11]. Side-effects are major determinants of compliance. By far, fever was also one of the most commonly observed side effects. In a retrospective review by Altamirano et al., of 10443 patients who were treated with furazolidone, approximately 0.34% experienced fever [12]. Drug fever accounts for 0.01%-5% of all adverse reactions and is a common condition that is frequently misdiagnosed [13,14]. A wide variety of agents can cause drug fever. However, antimicrobials are one of the most frequently caused fever drugs [15]. In the past few years, furazolidone has been chosen because of the lack of resistance of *H. pylori* against this drug, as well as its high efficacy [16,17]. However, its safety remains inconclusive; the incidence of drug fever in clinical findings was found to be much higher than that reported. Overall, the inclusion of furazolidone in a treatment regimen for infection does not absolutely appear to be safe.

In the present study, 1499 patients who received furazolidone treatment for *H. pylori* infection were analyzed to summarize the clinical features of drug fevers caused by furazolidone. The findings may help with the diagnosis and treatment of drug fever caused by furazolidone. Our goal is to increase awareness and stimulate further research on this topic.

2 Materials and Methods

2.1 Study design

This was a retrospective case-control study using the medical records of patients treated with furazolidone for *Helicobacter pylori* (*H. pylori*) infection at the Second Affiliated Hospital of Zhejiang University, School of Medicine (SAHZU), which is a tertiary care hospital located in Eastern China, with a total capacity of 3200 licensed beds. The enrolled participants had been infected between July 2018 and September 2018 and were all Han Chinese. Participants were all anonymous, and investigators had no access to patient information during or after data collection. The inclusion criteria selected (1) patients aged ≥18 years and (2) patients who received furazolidone treatment for *Helicobacter pylori* infection. The following patients were excluded: (1) patients who were pregnant or breastfeeding; (2) patients who received furazolidone treatment not for *Helicobacter pylori* infection; (3) patients taking antibiotics or any acid suppressant or non-steroidal anti-inflammatory drug in the last 4 weeks; and (4) patients with chronic hepatic, renal, or pulmonary disease.

2.2 Data Collection and Definitions

The possibility of a specific drug causing fever was evaluated by Naranjo algorithm [18]. Fever was defined as body temperature ≥38℃. It was concluded that fever had been caused by medication if it cleared rapidly after the suspected drug was discontinued [19]. We made a follow up on these patients for one month after they left hospital.

For each patient, the collected data included demographics such as age, gender, weight, and past drug allergy. We also collected data on the duration of the regimen and concomitant drugs (e.g., amoxicillin, clarithromycin, levofloxacin, colloidal bismuth pectin, bismuth potassium citrate, pantoprazole, rabeprazole, esomeprazole, and lansoprazole) with furazolidone therapy in patients with drug fever. In addition, white blood cells, neutrophils and eosnophils were obtained from the lab results.

2.3 Statistical analysis

Continuous variables are presented as the mean ± SD or median (interquartile range [IQR]) depending on whether they are normally distributed. Categorical variables are expressed as frequencies (%). Using Student's t-tests for independent variables and the Mann–Whitney U test for non-normally distributed data, we compared continuous variables. In contrast, Pearson's χ^2-test was used to compare categorical variables. Separate logistic regression analyses and multivariable logistic regression analyses were performed to determine risk factors associated with drug fever during anti-*Helicobacter pylori* therapy. Covariates were based on significant variables in the univariable model (i.e., $P < 0.05$). All statistical analyses were performed in SPSS for Windows version 22.0. $P < 0.05$ was considered statistically significant.

2.4 Ethics approval

This study was approved by the medical ethics committee of the Second Affiliated Hospital, Zhejiang University School of Medicine, China (No. 2020-283). The need for informed consent was waived due to the retrospective design of this study. All data collected was kept confidential.

3 Results

A total of 1503 patients were enrolled into the study. However, 4 patients were later excluded because they met the exclusion criteria, including received furazolidone treatment not for *Helicobacter pylori* infection (n=3), taking antibiotics or any acid suppressant or non-steroidal anti-inflammatory drug in the last 4 weeks (n=1). Eventually, 1499 patients were included, and 27 of these patients showed furazolidone drug fever. The incidence of furazolidone drug fever was as high as 1.80%. The average age of the adverse reaction group was 38.85±11.34 years old, and the other group was 39.94±12.66 years old. Notably, we found no differences in age and past drug allergy between the non-fever and fever groups. Among the 27 fever patients, 21 cases were female, accounting for 77.8%, which was significantly different between the two groups (*P*=0.005). The average body weight of the fever group was 57.52±7.60kg, and that of the non-fever group was 62.6±13.90kg, indicating a significant difference between the two groups (*P*=0.037) (Table 1). Concomitant medications, except for amoxicillin and clarithromycin (*P*=0.004 and *P*<0.001, respectively), were similar between the groups (Table 2).

Table 1. Demographic and clinical characteristics of patients with *Helicobacter pylori* infection.

Variable	Non-fever(n=1472)	Fever(n=27)	*P*
**Age, years**	39.94±12.66	38.85±11.34	0.237
**Gender, male**	718(48.8%)	6(22.2%)	0.005

**Gender, female**	754(51.2%)	21(77.8%)	0.005
**Weight, kg**	62.6±13.90	57.52±7.60	0.037
**Past drug allergy**	150(10.2%)	2(7.4%)	0.473

Data are presented as n (%) or mean ± SD

Table 2. Drug dosing and concomitant drugs in patients with and without drug fever.

**Furazolidone combined drugs**	**Non-fever(n=1472)**	**Fever(n=27)**	***P***
**Amoxicillin (1.0g bid)**	1256(85.3%)	17(63.0%)	**0.004**
**Clarithromycin (0.5g bid)**	152(10.3%)	10(37.0%)	**< 0.001**
**Levofloxacin (0.5g qd)**	39(2.6%)	0	0.488
**Colloidal bismuth pectin (0.2g bid)**	889(60.4%)	15(55.6%)	0.374
**Bismuth potassium citrate (0.6g bid)**	574(39.0%)	12(44.4%)	0.349
**Pantoprazole (40mg bid)**	336(22.8%)	4(14.8%)	0.232
**Rabeprazole (10mg bid)**	807(54.8%)	19(70.4%)	0.077
**Esomeprazole (20mg bid)**	201(13.7%)	3(11.1%)	0.488
**Lansoprazole (30mg bid)**	121(8.2%)	2(7.4%)	0.616

Data are presented as n (%)

As shown in Table 3, the mean time between the initiation of furazolidone and the onset of fever is 11.00±1.84 days, and the median peak fever was 38.87±0.57℃. The leukocyte count and eosinophil level were both normal. The neutrophil mean count was (8.34±3.57) × 10^9/L, which was slightly elevated in this research.

Table 3. Duration of regimens and laboratory tests of patients with observed fever.

Laboratory Tests	Mean	Normal Ranges
**Duration of regimen (days)**	11.00±1.84	
**Body temperature (℃)**	38.87±0.57	36.0~37.0
**White blood cells (×10^9/L)**	9.84±3.63	4.0~10.0
**Neutrophils (×10^9/L)**	8.34±3.57	2.0~7.0
**Eosnophils (×10^9/L)**	0.18±0.15	0.0~1.0

Data are presented as mean ± SD

Univariate analysis revealed four variables related to drug fever, including gender, weight, amoxicillin, and clarithromycin. Furthermore, we revealed two independent variables to be associated with the onset of drug fever after adjusting the underlying confounders through multivariate analysis. These variables included gender (OR, 3.162; 95% CI, 1.264-7.914; P=0.014) and clarithromycin (OR, 4.834; 95% CI, 2.165–10.794; P<0.001) (Table 4).

Table 4. Univariable and multivariable logistic regression analysis for independent risk factors for furazolidone-associated fever in patients.

Risk factor	Unadjusted OR (95% CI)	***P***	Adjusted OR (95% CI)	***P***
**Gender**	3.333(1.338-8.305)	0.010	3.162(1.264-7.914)	0.014
**Weight**	0.967(0.937-0.999)	0.045		
**Amoxicillin**	0.292(0.132-0.647)	0.002		
**Clarithromycin**	5.108(2.298-11.357)	< 0.001	4.834(2.165-10.794)	< 0.001

OR, odd ratio; 95% CI, 95% confidence interval

4 Discussion

Although known as a rare adverse drug reaction (ADR), drug fever remains an important issue in medicine, with the risk of leading to inappropriate and potentially harmful diagnostic and therapeutic interventions. Only sparse data regarding drug fever have been published. In this case series, we present 27 (1.80%) patients with drug fever out of 1499 patients administered furazolidone during a three-month period in our hospital. Furazolidone is a synthetic nitrofuran with a broad spectrum of antimicrobial action and has been widely used in the treatment of gastrointestinal infections [10]. The side effects of furazolidone-containing therapies have varied across different studies, and they occur more frequently for these therapies than standard therapies, which include nausea, vomiting, diarrhea, drug fever, skin rash, and so on [20,21]. In this study, the incidence of drug fever caused by furazolidone was 1.80%, which was higher than 0.34%, which was reported in previous assessments [12]. Drug fever may occur at any point during a course of drug therapy, and there is significant variation among different drug classes. Drug fever most commonly appears after 7-10 days of drug administration [14]. In this study, the average cumulative medication days of furazolidone in fever patients was 11.00±1.84 days, which was slightly longer than other antibacterial drugs. According to Johnson D.H. et al., laboratory findings can be helpful in supporting a diagnosis of drug fever, although they are highly variable and cannot be relied on for a definitive diagnosis [22]. Leukocytosis with or without a left shift may be present. In

all patients with suspected drug fever in our study, the leukocyte count was within the normal range. Eosinophil levels are frequently elevated as reported by others, while we found that the absolute value of eosinophils in patients with suspected drug fever was not affected. No other laboratory tests were measured in this research. After furazolidone discontinuation, the body temperature returned to normal after 48 h. Furazolidone interferes with the activity of bacterial oxidoreductase, blocking bacterial metabolism. It is a monoamine oxidase inhibitor (MAOI) that can interact with a number of drugs and commonly used foods such as soy sauce, aged cheese, and beer [23]. If a patient eats foods that contain tyramine while taking furazolidone, sympathetic nerves are easily excited. These side-effects can probably be prevented by avoiding tyramine-containing foods. When taking furazolidone, a small amount of tea or coffee can cause insomnia, and a large amount of tea can lead to high blood pressure. It is generally believed that tea or coffee drinking should be prohibited two hours before taking furazolidone.

To the best of our knowledge, this is the first report on the independent risk factors for furazolidone-associated drug fever. In this single-center study evaluating furazolidone-based quadruple therapy for *H. pylori* infection, the dosage of furazolidone was 0.1 g, b.i.d., according to the drug instruction. In addition, Roghani et al. reported that fever, fatigue, and dizziness were more common in high-dose furazolidone-containing therapies (200 mg, b.i.d.) than low-dose therapies (50 mg, b.i.d.) [24]. These results indicate that the side-effects of furazolidone are associated with dose. While there was no difference in furazolidone dose (100 mg, b.i.d.) between

the two groups in our study, we cannot determine whether furazolidone dose influenced drug fever. Other investigated risk factors including age, weight, past drug allergy, and concomitant administration for *H. pylori* infection, except for clarithromycin, were similar between the two groups. Retrospectively, we noted that the drug fever of furazolidone was significantly associated with gender (female versus male, $P = 0.005$). According to Lipsky et al. [25], the risk of drug fever in female patients is significantly increased, which is consistent with the results of this study. A highlight of this study was that clarithromycin was revealed to be an independent risk factor for drug fever. In this study, the combination of furazolidone and amoxicillin was not prone to drug fever, but the combined use of clarithromycin was more prone to drug fever. The inhibition of hepatic cytochrome P-450 enzymes by clarithromycin has been well documented [26]. The mechanism of inhibition probably involves the induction of demethylation and oxidation by cytochrome P-450 of the tertiary amine on the amino sugar of the macrolide to its nitroso intermediate, which then forms an inactive complex with the iron of cytochrome P-450. Reported drug interactions include enhanced glucocorticoid effects, theophylline toxicity, carbamazepine toxicity, and the clinical failure of oral contraceptives [27]. Whether it will affect the metabolism of furazolidone has not been reported yet, and further studies are needed.

This study suffers from the following limitations. Firstly, it was a retrospective single-center study with a small sample size. Our 27 patients represent a convenience sample; the true incidence of furazolidone-associated drug fever cannot be determined with certainty. Therefore, we could not examine the contribution of all possible risk

factors. Secondly, drug fever may have been induced by multifactorial causes besides furazolidone. Although we adjusted various factors related to drug fever, unadjusted factors may still have affected the development of fever. Thirdly, as all patients were outpatients in this study, there were very few laboratory values on them. We could not collect enough data due to the limitations of our design. This should be considered in further investigations.

In summary, we described 27 patients infected with *H. pylori* with the onset of clinically relevant hyperthermia during furazolidone administration, highly suggestive of drug fever. We also identified being female and the combined use of clarithromycin as risk factors for furazolidone-induced drug fever. Drug fever is a common and potentially serious adverse reaction to furazolidone of which clinicians should have increased awareness. As the underlying mechanisms are uncertain to date, further research on this topic is warranted.

Acknowledgments

We thank Linxiao Dai, Xiuping Zhu, Huan Luo, Yuwen Huang, Wei He, Junfeng Li for their significant help in this work.

Author Contributions

Conceptualization: Jiali Zhang, Haibin Dai.

Data curation: Jiali Zhang, Chunling Rong, Chenyang Yan.

Formal analysis: Jie Chen.

Funding acquisition: Jiali Zhang, Lingyan Yu.

Investigation: Jiali Zhang,

Methodology: Jie Chen, Wenjun Yang, Lingyan Yu.

Supervision: Haibin Dai.

Validation: Haibin Dai.

Writing - original draft: Jiali Zhang.

Writing - review & editing: Chunling Rong, Chenyang Yan, Jie Chen, Wenjun Yang, Lingyan Yu.

Funding

This project was supported by a grant from the Natural Science Foundation of Zhejiang Province (Grant No. LYY19H310013), the Foundation from the Health Bureau of Zhejiang Province (Grant No. 2019KY406), and the foundation from Zhejiang Pharmaceutical Association (Grant No. 2019ZYY18).

References:

1. Hooi JKY, Lai WY, Ng WK, Suen MMY, Underwood FE, Tanyingoh D, et al. Global Prevalence of Helicobacter pylori Infection: Systematic Review and Meta-Analysis. Gastroenterology. 2017;153: 420–429. doi:10.1053/j.gastro.2017.04.022
2. Nonghua Lu LZ. Guideline for primary care of Helicobacter pylori infection(2019). Chin J Gen Pr. 2020;19: 397–402.

doi:10.3760/cma.j.cn114798

3. Savoldi A, Carrara E, Graham DY, Conti M, Tacconelli E. Prevalence of Antibiotic Resistance in Helicobacter pylori: A Systematic Review and Meta-analysis in World Health Organization Regions. Gastroenterology. 2018;155: 1372-1382.e17. doi:10.1053/j.gastro.2018.07.007

4. Hu Y, Zhu Y, Lu N hua. Primary Antibiotic Resistance of Helicobacter pylori in China. Dig Dis Sci. 2017;62: 1146–1154. doi:10.1007/s10620-017-4536-8

5. Mokhtare M, Hosseini V, Fakheri HT, Maleki I, Taghvaei T, Valizadeh SM, et al. Comparison of quadruple and triple Furazolidone containing regimens on eradication of helicobacter pylori. Med J Islam Repub Iran. 2015;29.

6. Hosseini V, Mokhtare M, Gholami M, Taghvaei T, Maleki I, Valizadeh M, et al. A Comparison between Moderate- and High-dose Furazolidone in Triple Regimens for Helicobacterpylori Eradication in Iran. Middle East J Dig Dis. 2014;6: 195-202–202. doi:10.15171/middleeastjdi.v6i4.1347

7. Cheng H, Hu FL. Furazolidone, amoxicillin, bismuth and rabeprazole quadruple rescue therapy for the eradication of Helicobacter pylori. World J Gastroenterol. 2009;15: 860–864. doi:10.3748/wjg.15.860

8. Liu WZ, Xie Y, Lu H, Cheng H, Zeng ZR, Zhou LY, et al. Fifth Chinese National Consensus Report on the management of Helicobacter pylori infection. Helicobacter. 2018;23: 346–360. doi:10.1111/hel.12475

9. Mohammadi M, Attaran B, Malekzadeh R, Graham DY. Furazolidone, an Underutilized Drug for H. pylori Eradication: Lessons from Iran. Dig Dis Sci.

2017;62: 1890–1896. doi:10.1007/s10620-017-4628-5

10. Buzás GM, Józan J. Nitrofuran-based regimens for the eradication of Helicobacter pylori infection. J Gastroenterol Hepatol. 2007;22: 1571–1581. doi:10.1111/j.1440-1746.2007.05082.x

11. Fakheri H, Malekzadeh R, Merat S, Khatibian M, Fazel A, Alizadeh BZ, et al. Clarithromycin vs. furazolidone in quadruple therapy regimens for the treatment of Helicobacter pylori in a population with a high metronidazole resistance rate. Aliment Pharmacol Ther. 2001;15: 411–416. doi:10.1046/j.1365-2036.2001.00931.x

12. Altamirano A, Bondani A. Adverse reactions to furazolidone and other drugs: A comparative review. Scand J Gastroenterol. 1989;24: 70–80. doi:10.3109/00365528909091336

13. Fang Y, Xiao H, Tang S, Liang L, Sha W, Fang Y. Clinical features and treatment of drug fever caused by anti-tuberculosis drugs. Clin Respir J. 2016;10: 449–454. doi:10.1111/crj.12242

14. Patel RA, Gallagher JC. Drug fever. Pharmacotherapy. 2010;30: 57–69. doi:10.1592/phco.30.1.57

15. Dominique Vodovar, Christine LeBeller, Bruno Me´garbane AL-L-L and TH. Drug fever. Drug Saf. 2012;35: 759–767. doi:10.1016/S0891-5520(05)70287-7

16. Zhang YW, Hu WL, Cai Y, Zheng WF, Du Q, Kim JJ, et al. Outcomes of furazolidone-and amoxicillin-based quadruple therapy for Helicobacter pylori infection and predictors of failed eradication. World J Gastroenterol. 2018;24:

4596–4605. doi:10.3748/wjg.v24.i40.4596

17. Xie Y, Zhang Z, Hong J, Liu W, Lu H, Du Y, et al. Furazolidone-containing triple and quadruple eradication therapy for initial treatment for Helicobacter pylori infection: A multicenter randomized controlled trial in China. Helicobacter. 2018;23: 1–7. doi:10.1111/hel.12496

18. Naranjo CA, Busto U, Sellers EM, Sandor P, Ruiz I, Roberts EA, et al. A method for estimating the probability of adverse drug reactions. Clin Pharmacol Ther. 1981;30: 239–245. doi:10.1038/clpt.1981.154

19. Hung YP, Lee NY, Chang CM, Lee HC, Wu CJ, Chen PL, et al. Tolerability of teicoplanin in 117 hospitalized adults with previous vancomycin-induced fever, rash, or neutropenia: A retrospective chart review. Clin Ther. 2009;31: 1977–1986. doi:10.1016/j.clinthera.2009.09.010

20. De Francesco V, Ierardi E, Hassan C, Zullo A. Furazolidone therapy for Helicobacter pylori: Is it effective and safe? World J Gastroenterol. 2009;15: 1914–1915. doi:10.3748/wjg.15.1914

21. Song C, Qian X, Zhu Y, Shu X, Song Y, Xiong Z, et al. Effectiveness and safety of furazolidone-containing quadruple regimens in patients with Helicobacter pylori infection in real-world practice. Helicobacter. 2019;24: 1–12. doi:10.1111/hel.12591

22. Johnson DH, Cunha BA. Drug fever. Infect Dis Clin North Am. 1996;10: 85–91. doi:10.1016/S0891-5520(05)70287-7

23. McCabe-Sellers BJ, Staggs CG, Bogle ML. Tyramine in foods and monoamine

oxidase inhibitor drugs: A crossroad where medicine, nutrition, pharmacy, and food industry converge. J Food Compos Anal. 2006;19: 58–65. doi:10.1016/j.jfca.2005.12.008

24. Roghani HS, Massarrat S, Shirekhoda M, Butorab Z. Effect of different doses of furazolidone with amoxicillin and omeprazole on eradication of Helicobacter pylori. J Gastroenterol Hepatol. 2003;18: 778–782. doi:10.1046/j.1440-1746.2003.03058.x

25. Lipsky BA, Hirschmann J V. Drug Fever. JAMA J Am Med Assoc. 1981;245: 851–854. doi:10.1001/jama.1981.03310330041024

26. Sturgill MG, Rapp RP. Clarithromycin: review of a new macrolide antibiotic with improved microbiologic spectrum and favorable pharmacokinetic and adverse effect porfiles. Ann Pharmacother. 1992;26: 1099–1108.

27. Kuper JJ, D’Aprile M. Drug-drug interactions of clinical significance in the treatment of patients with Mycobacterium avium complex disease. Clin Pharmacokinet. 2000;39: 203–214. doi:10.2165/00003088-200039030-00003
