# Supplementary material for: Risk factors of furazolidone-associated fever
Source: PLoS One. 2022 Apr 8;17(4):e0266763. doi: 10.1371/journal.pone.0266763 (PMC8993017; doi:10.1371/journal.pone.0266763)
Supplement: Attachment — Submitted filename: Response to Reviewers.docx [file pone.0266763.s002.docx]

Thank you for your letter and for the reviewers' comments concerning our manuscript entitled “Risk Factors of Furazolidone-Associated Fever”. These comments are all valuable and very helpful for revising and improving our paper, as well as the important guiding significance to our research. We have studied comments carefully and have made corrections which we hope to meet with approval. The responds to the editor and reviewers' comments are as follows:

**Journal Requirements:**

1. Please ensure that your manuscript meets PLOS ONE's style requirements, including those for file naming. The PLOS ONE style templates can be found at

<https://journals.plos.org/plosone/s/file?id=wjVg/PLOSOne_formatting_sample_main_body.pdf>  and <https://journals.plos.org/plosone/s/file?id=ba62/PLOSOne_formatting_sample_title_authors_affiliations.pdf>

**Response:** Thank you for your suggestion. We have read the PLOS ONE style templates and modified one by one according to it.

2. We note that you have indicated that data from this study are available upon request. PLOS only allows data to be available upon request if there are legal or ethical restrictions on sharing data publicly. For more information on unacceptable data access restrictions, please see <http://journals.plos.org/plosone/s/data-availability#loc-unacceptable-data-access-restrictions>.

In your revised cover letter, please address the following prompts:

a) If there are ethical or legal restrictions on sharing a de-identified data set, please explain them in detail (e.g., data contain potentially sensitive information, data are owned by a third-party organization, etc.) and who has imposed them (e.g., an ethics committee). Please also provide contact information for a data access committee, ethics committee, or other institutional body to which data requests may be sent.

b) If there are no restrictions, please upload the minimal anonymized data set necessary to replicate your study findings as either Supporting Information files or to a stable, public repository and provide us with the relevant URLs, DOIs, or accession numbers. For a list of acceptable repositories, please see http://journals.plos.org/plosone/s/data-availability#loc-recommended-repositories.

**Response:** Thank you for your suggestion. The data that support the findings of this study contain potentially sensitive patient information. Restrictions apply to the availability of these data, which were used under license for this study. Data are available from corresponding author Haibin Dai with the permission of the Ethics Committee of the 2^nd^ Affiliated Hospital, School of Medicine, Zhejiang University.

We have revised cover letter and explain the reasons of ethical restrictions on sharing a de-identified data set. We also provide contact information which data requests may be sent in the revised cover letter.

3. Thank you for stating the following in the Funding Section of your manuscript:

“This project was supported by a grant from the Natural Science Foundation of Zhejiang Province (Grant No. LYY19H310013), the Foundation from the Health Bureau of Zhejiang Province (Grant No. 2019KY406), and the foundation from Zhejiang Pharmaceutical Association (Grant No. 2019ZYY18).”

We note that you have provided additional information within the Funding Section. Please note that funding information should not appear in other areas of your manuscript. We will only publish funding information present in the Funding Statement section of the online submission form.

Please remove any funding-related text from the manuscript and let us know how you would like to update your Funding Statement. Currently, your Funding Statement reads as follows:

“Yes, this project was supported by a grant from the Natural Science Foundation of Zhejiang Province (Grant No. LYY19H310013) which was received by JL Zhang, the Foundation from the Health Bureau of Zhejiang Province (Grant No. 2019KY406) which was received by JL Zhang, and the foundation from Zhejiang Pharmaceutical Association (Grant No. 2019ZYY18) which was received by LY Yu.”

Please include your amended statements within your cover letter; we will change the online submission form on your behalf.

**Response:** Thank you for your suggestion. We have removed the funding-related text from the manuscript. My funding statement is the same as before: “This project was supported by a grant from the Natural Science Foundation of Zhejiang Province (Grant No. LYY19H310013) which was received by JL Zhang, the Foundation from the Health Bureau of Zhejiang Province (Grant No. 2019KY406) which was received by JL Zhang, and the foundation from Zhejiang Pharmaceutical Association (Grant No. 2019ZYY18) which was received by LY Yu.”

4. Thank you for stating the following in the Competing Interests/Financial Disclosure * (delete as necessary) section:

“This project was supported by a grant from the Natural Science Foundation of Zhejiang Province (Grant No. LYY19H310013), the Foundation from the Health Bureau of Zhejiang Province (Grant No. 2019KY406), and the foundation from Zhejiang Pharmaceutical Association (Grant No. 2019ZYY18).”

We note that you received funding from a commercial source: Zhejiang Pharmaceutical Association

Please provide an amended Competing Interests Statement that explicitly states this commercial funder, along with any other relevant declarations relating to employment, consultancy, patents, products in development, marketed products, etc.

Within this Competing Interests Statement, please confirm that this does not alter your adherence to all PLOS ONE policies on sharing data and materials by including the following statement: "This does not alter our adherence to PLOS ONE policies on sharing data and materials.” (as detailed online in our guide for authors http://journals.plos.org/plosone/s/competing-interests). If there are restrictions on sharing of data and/or materials, please state these. Please note that we cannot proceed with consideration of your article until this information has been declared.

Please include your amended Competing Interests Statement within your cover letter. We will change the online submission form on your behalf.

**Response:** Thank you for your suggestion. Zhejiang Pharmaceutical Association is a none profit organization in China. The founder from Zhejiang Pharmaceutical Association is also not a commercial funder. We adhere PLOS ONE policies on sharing data and materials all the time.

**Reviewers' comments:**

1. Is the manuscript technically sound, and do the data support the conclusions?

The manuscript must describe a technically sound piece of scientific research with data that supports the conclusions. Experiments must have been conducted rigorously, with appropriate controls, replication, and sample sizes. The conclusions must be drawn appropriately based on the data presented.

Reviewer #1: Yes

Reviewer #2: Partly

**Response:** Yes, our manuscript described a technically sound piece of scientific research. The research has been conducted rigorously, with appropriate controls and sample sizes. This retrospective cohort study identified two risk factors for furazolidone-associated fever, which were female and clarithromycin. We also analyzed the characteristics of drug fever during anti-*Helicobacter pylori* therapy. These conclusions were drawn appropriately based on the data presented.

2. Has the statistical analysis been performed appropriately and rigorously?

Reviewer #1: Yes

Reviewer #2: I Don't Know

**Response:** Yes, the statistical analysis has been performed appropriately and rigorously with SPSS for Windows version 22.0 in our manuscript.

3. Have the authors made all data underlying the findings in their manuscript fully available?

The PLOS Data policy requires authors to make all data underlying the findings described in their manuscript fully available without restriction, with rare exception (please refer to the Data Availability Statement in the manuscript PDF file). The data should be provided as part of the manuscript or its supporting information, or deposited to a public repository. For example, in addition to summary statistics, the data points behind means, medians and variance measures should be available. If there are restrictions on publicly sharing data—e.g. participant privacy or use of data from a third party—those must be specified.

Reviewer #1: Yes

Reviewer #2: No

**Response:** Yes, the data that support the findings of this study contain potentially sensitive patient information. Data are available from corresponding author Haibin Dai with the permission of the Ethics Committee of the 2^nd^ Affiliated Hospital, School of Medicine, Zhejiang University.

4. Is the manuscript presented in an intelligible fashion and written in standard English?

PLOS ONE does not copyedit accepted manuscripts, so the language in submitted articles must be clear, correct, and unambiguous. Any typographical or grammatical errors should be corrected at revision, so please note any specific errors here.

Reviewer #1: Yes

Reviewer #2: No

**Response:** Yes, the manuscript has been thoroughly revised and edited by a native speaker. The certificate provided by multidisciplinary digital publishing institute was in the attached document. We really hope that the language level have been substantially improved.

**Reviewer #1:**

The underlying mechanisms of furazolidone causing fever should be discussed.

**Response:** Thank you for your suggestion. We have added the following discussion to further explain the underlying mechanisms of furazolidone causing fever in revised manuscript as follow: “Line#201-211 However, the exact mechanisms of furazolidone causing fever are remain undetermined. It is chemically related to nitrofurantoin, which is well known to cause pleuropneumonic reactions [25]. These acute hypersensitivity reactions typically begin with fever, cough, and dyspnea [26]. The proposed mechanisms involve a cytotoxic response, an immune-complex mediated response and a cell mediated reaction [27]. The mechanism of pyrexia in cellular immunity appears to be due to production of nonpyrogenic soluble factors that act on macrophages to produce endogenous pyrogen to result in fever [15]. On the other hand, as early as the 1960s, furazolidone was shown to be an inhibitor of monoamine oxidase activity. It can interact with a number of drugs and commonly used foods such as soy sauce, aged cheese, and beer [28].”

Line#64 However, the resistance rates of other antibiotics, such as tetracycline and furazolidone, are still low. Reference is missing.

**Response:** Thank you for your suggestion. We have cited the reference “However, the resistance rates of other antibiotics, such as tetracycline and furazolidone, are still low [5].”

Line#77 A wide variety of agents can cause drug fever Reference is missing.

**Response:** Thank you for your suggestion. We have cited the reference “A wide variety of agents have been shown to induce drug fever [15].”

Line# 82 Overall, the inclusion of furazolidone in a treatment regimen for infection does not absolutely appear to be safe. Reference is missing.

**Response:** Thank you for your suggestion. We have cited the reference “Overall, the inclusion of furazolidone in a treatment regimen for infection does not absolutely appear to be safe [19].”

How the authors conducted a follow up on the study patients for one month after they left hospital?

**Response:** Thank you for your suggestion. Patients were contacted by telephone at 0.5 and 1 months after they left hospital by pharmacists who were blinded to the grouping.

The average age of the adverse reaction group was 38.85±11.34 years old, and the other group was 39.94±12.66 years old. The sentence is NOT clear what is the other group?

**Response:** Thank you for your suggestion. We are very sorry for the inconvenience it caused in your reading. We have changed the sentence “The average age of the adverse reaction group was 38.85±11.34 years old, and the other group was 39.94±12.66 years old” to “The average age of the adverse reaction group was 38.85±11.34 years old, and the non-fever group was 39.94±12.66 years old” in the revised manuscript.

**Reviewer #2:**

Explain the exclusion criteria; for) patients were pregnant or breastfeeding- as being the high risk population why they were excluded

**Response:** Thank you for your suggestion. Furazolidone is contraindicated in the dispensatory for pregnant and breastfeeding women in China. Furazolidone used by pregnant or breastfeeding patients is an off-label use. However, none of the 4 patients excluded in this study were pregnant or breastfeeding women.

in abstract - result section mentioned -A total of 1499 patients received polymyxin and met the overall inclusion criterion- may be had to write Furazolidone and by mistake mentioned polymyxin - needs correction

**Response:** We are extremely grateful to you for pointing out this problem. We have changed this sentence to “A total of 1499 patients received furazolidone and met the overall inclusion criterion”.

Drug response can be impacted by several factors including diet, comorbidities, age, weight, drug–drug interactions, and genetics. Individual genetic variation in key genes involved in the metabolism, transport, or drug target can contribute to risk of adverse events or treatment failure. I feel very little interest while reading this paper as very important aspects are missing.

**Response:** Thank you for your suggestion. We agree that drug response can be impacted by several factors. In fact, our research is the first to show the independent risk factors for furazolidone-associated drug fever. We confirm the findings of being female and the combined use of clarithromycin as risk factors for furazolidone-induced drug fever. At present, individual genetic variation in key genes involved in the metabolism, transport, or drug target can contribute to the risk of furazolidone-associated drug fever is still unclear. We want to do such studies in the future. At present, our findings can improve furazolidone-treated patients' safety to some extent. We also have added limitation in the Discussion section to clarify this in revised manuscript as follow: “Line#246-251 Secondly, drug fever may have been induced by multifactorial causes including diet, comorbidities, age, weight, drug–drug interactions, and genetics. Individual genetic variation in key genes involved in the metabolism, transport, or drug target can contribute to the risk of adverse events. Although we have examined various factors related to furazolidone-associated drug fever, some other risk factors remain unexamined which may affect this adverse event.”

The manuscript should be checked by a native speaker in terms of language and grammar. I highlighted only some passages in the attached document.

**Response:** We apologize for the poor language of our manuscript. All the highlighted passages in the attached document have been revised. The manuscript has been thoroughly revised and edited by a native speaker. The certificate provided by multidisciplinary digital publishing institute was in the attached document. We really hope that the language level have been substantially improved.

I am not sure for PLOS ONE criteria for manuscript selection but the finding of the paper are not significant due to lack of data about very important variables - that can address this topic clearly

**Response:** Thank you for your suggestion. We agree that drug response can be impacted by several factors. In fact, our research is the first to show the independent risk factors for furazolidone-associated drug fever. We confirm the findings of being female and the combined use of clarithromycin as risk factors for furazolidone-induced drug fever. At present, our findings can improve furazolidone-treated patients' safety to some extent. As the underlying mechanisms are uncertain to date, we will do further research on this topic in the future.

Best wishes,

Yours sincerely ,

Haibin Dai
