# Supplementary material for: Risk factors of furazolidone-associated fever
Source: PLoS One. 2022 Apr 8;17(4):e0266763. doi: 10.1371/journal.pone.0266763 (PMC8993017; doi:10.1371/journal.pone.0266763)
Supplement: Attachment — Submitted filename: Response to Reviewers.docx [file pone.0266763.s003.docx]

Thank you for your letter and for the reviewers' comments concerning our manuscript entitled “Risk Factors of Furazolidone-Associated Fever”. These comments are all valuable and very helpful for revising and improving our paper, as well as the important guiding significance to our research. We have studied comments carefully and have made corrections which we hope to meet with approval. The responds to the editor and reviewers' comments are as follows:

Reviewers' comments:

Reviewer's Responses to Questions

**Comments to the Author**

1. If the authors have adequately addressed your comments raised in a previous round of review and you feel that this manuscript is now acceptable for publication, you may indicate that here to bypass the “Comments to the Author” section, enter your conflict of interest statement in the “Confidential to Editor” section, and submit your "Accept" recommendation.

2. Is the manuscript technically sound, and do the data support the conclusions?

The manuscript must describe a technically sound piece of scientific research with data that supports the conclusions. Experiments must have been conducted rigorously, with appropriate controls, replication, and sample sizes. The conclusions must be drawn appropriately based on the data presented.

Reviewer #1: Yes

Reviewer #2: Yes

Reviewer #3: No

**Response:** Yes, our manuscript described a technically sound piece of scientific research. The research has been conducted rigorously, with appropriate controls and sample sizes. This retrospective cohort study identified two risk factors for furazolidone-associated fever, which were female and clarithromycin. We also analyzed the characteristics of drug fever during anti-*Helicobacter pylori* therapy. These conclusions were drawn appropriately based on the data presented.

3. Has the statistical analysis been performed appropriately and rigorously?

Reviewer #1: Yes

Reviewer #2: Yes

Reviewer #3: No

**Response:** Yes, the statistical analysis has been performed appropriately and rigorously with SPSS for Windows version 22.0 in our manuscript.

4. Have the authors made all data underlying the findings in their manuscript fully available?

The [PLOS Data policy](http://www.plosone.org/static/policies.action#sharing) requires authors to make all data underlying the findings described in their manuscript fully available without restriction, with rare exception (please refer to the Data Availability Statement in the manuscript PDF file). The data should be provided as part of the manuscript or its supporting information, or deposited to a public repository. For example, in addition to summary statistics, the data points behind means, medians and variance measures should be available. If there are restrictions on publicly sharing data—e.g. participant privacy or use of data from a third party—those must be specified.

Reviewer #1: Yes

Reviewer #2: (No Response)

Reviewer #3: No

**Response:** Yes, the data that support the findings of this study contain potentially sensitive patient information. Data are available from corresponding author Haibin Dai with the permission of the Ethics Committee of the 2^nd^ Affiliated Hospital, School of Medicine, Zhejiang University.

5. Is the manuscript presented in an intelligible fashion and written in standard English?

PLOS ONE does not copyedit accepted manuscripts, so the language in submitted articles must be clear, correct, and unambiguous. Any typographical or grammatical errors should be corrected at revision, so please note any specific errors here.

Reviewer #1: Yes

Reviewer #2: Yes

Reviewer #3: No

**Response:** Yes, the manuscript has been thoroughly revised and edited by a native speaker. The certificate provided by multidisciplinary digital publishing institute was in the attached document. We really hope that the language level has been substantially improved.

6. PLOS authors have the option to publish the peer review history of their article ([what does this mean?](https://journals.plos.org/plosone/s/editorial-and-peer-review-process#loc-peer-review-history)). If published, this will include your full peer review and any attached files.

If you choose “no”, your identity will remain anonymous but your review may still be made public.

**Do you want your identity to be public for this peer review?** For information about this choice, including consent withdrawal, please see our [Privacy Policy](https://www.plos.org/privacy-policy).

Reviewer #1: No

Reviewer #2: **Yes:** Dr. Gul Ambreen

Reviewer #3: No

7. Review Comments to the Author

Please use the space provided to explain your answers to the questions above. You may also include additional comments for the author, including concerns about dual publication, research ethics, or publication ethics. (Please upload your review as an attachment if it exceeds 20,000 characters)

Reviewer #3: 1) Data collection procedures are not clear. Which data are from medical records of patients and which are gathered from telephone calls? Did the researchers make phone calls to the 1499 patients? How was Naranja algorithm used in the study? The results of the Naranja algorithm were not shown, the researchers only kept saying about fever getting lost when the drug is discontinued. How did the researchers know that fever was lost after 48 hrs? The incidence of drug fever caused by furazolidone was 1.80% according to the article but data to prove that these are furazolidone-induced fever cases are lacking. The increase in neutrophils in the lab results of the patients was not also explained, as this could be a reason also for the fever.

**Response:** We feel sorry for the inconvenience brought to the reviewer. Most data in this study, for example demographic and clinical characteristics of patients, drug dosing, concomitant drugs and laboratory tests were all from medical records. The time when fever lost was gathered from telephone calls. 27 Patients with drug fever who went back to hospital for further consultation were contacted by telephone at 0.5 and 1 months after the first time of they left hospital by pharmacists. However, other 1472 patients were only contacted by telephone at about 1 month after they left hospital.

We feel sorry for the results of the Naranja algorithm were not shown at first. We have supplemented its method in revised manuscript as follow: “Line#109-113 Two independent reviewers gave each suspected patients with drug fever a Naranjo scale score. The scores were averaged and rounded up to the higher integer. The final score interpretations were stratified into four categories with a score of ≥ 9 considered “definite”, 5 to 8 “probable”, 1 to 4 “possible”, and those ≤ 0 “doubtful” likelihood of the drug causing the ADR”. The results of the Naranja algorithm we have supplemented in revised manuscript as follow: “Line#146-147 The Naranjo score classified 0 (0.0%) of cases as definite, 25 (92.6%) probable, 2 (7.4%) possible, and 0 (0.0%) doubtful (Table 1)”. We also have supplemented the “Table 1” in line#158 as follow:

**Table 1. Naranjo score of patients with observed fever.**

| **Naranjo score** | **N (%)** |
| --- | --- |
| **Definite (≥ 9)** | 0 (0.0) |
| **Probable (5-8)** | 25 (92.6) |
| **Possible (1-4)** | 2 (7.4) |
| **Doubtful (≤ 0)** | 0 (0.0) |

A total of 1499 patients received furazolidone and met the overall inclusion criterion. Of these 1499 patients, 27 (1.80%) developed drug fever. We have supplemented the results of the Naranja algorithm in the revised manuscript. The Naranjo scale was used to assess the relationship between the adverse drug reactions and furazolidone therapy. Most of the score suggested a probable relationship.

The increase in neutrophils in the lab results of the patients could be influenced by many factors, for example infection, allergy, cancer and so on. When two independent reviewers gave each suspected patients with drug fever a Naranjo scale score have considered it. Thank you for your suggestion.

2) Mean onset of fever is 11 days, how about treatment duration less than 5 days or over 14 days, where they included in the 1499 patients enrolled?

**Response:** Thank you for your suggestion. Our study was a retrospective case-control study of 1499 patients who used furazolidone-containing quadruple regimens for *H. pylori* infection. All of them took therapeutic regimen for 14 days. Of these 1499 patients, 27 patients developed drug fever. We are very sorry for haven’t stated clearly in the original version. We have revised line#93-96 to “This was a retrospective case-control study using the medical records of patients treated with 14-day Furazolidone-containing quadruple regimens for *Helicobacter pylori* (*H. pylori*) infection at the Second Affiliated Hospital of Zhejiang University, School of Medicine (SAHZU)”.

3) The number of patients with furazolidone-induced fever in the study is 27, is this statistically enough to determine the risk factors? Did you compute the number of subjects with furazolidone-induced fever needed to determine risk factors for it? Statistical analysis to show that the risk factors for furazolidone-associated fever that were female and clarithromycin were not sufficient in the study. Such as, your basis of selecting females as a risk factor compared to males is that there are 21 females and 6 males out of the 27 had the furazolidone-induced fever. There are actually more females in the 1499 enrolled patients in the study. The basis for concluding clarithromycin as a risk factor was not well discussed and was not statistically validated.

**Response:** Thank you for your suggestion. In fact, this was a retrospective non-interventional case-control study, the gender of enrolled patients was based on actual medical events. We also have used the tests for the odds ratio in a matched case-control design with a binary X in PASS 2021. 27 patients with furazolidone-induced fever were statistically enough to determine the risk factors. The statistical reports provided by PASS 2021 was in the attached document.

We feel sorry for concluding clarithromycin as a risk factor was not well discussed. As we know, the clinical characteristics of patients and concomitant medication are common risk factors of ADR. Clarithromycin is one of the concomitant drugs in enrolled patients. So, we used separate logistic regression analyses and multivariable logistic regression analyses to determine whether clarithromycin associated with drug fever in this study. Covariates were based on significant variables in the univariable model (i.e., *P* < 0.05). The result of separate logistic regression analyses was showed in table 2 that concomitant medications, except for amoxicillin and clarithromycin (*P*=0.004 and *P*<0.001, respectively), were similar between the patients with or without furazolidone-associated fever. The result of multivariable logistic regression analyses was showed in table 5 that two independent variables to be associated with the onset of drug fever after adjusting the underlying confounders through multivariate analysis. These variables included gender (OR, 3.162; 95% CI, 1.264-7.914; *P*=0.014) and clarithromycin (OR, 4.834; 95% CI, 2.165–10.794; *P*<0.001).

The data collection procedure was not clear. The statistical analysis and data provided to support the conclusion of the study are not sufficient.

**Response:** We feel sorry for the inconvenience brought to the reviewer. We have declared the data collection procedure and statistical analysis in questions 1 and 3 at above. We also have revised our manuscript try my best. Thank you for your suggestion.

Best wishes,

Yours sincerely,

Haibin Dai
