# Supplementary material for: Risk factors of furazolidone-associated fever
Source: PLoS One. 2022 Apr 8;17(4):e0266763. doi: 10.1371/journal.pone.0266763 (PMC8993017; doi:10.1371/journal.pone.0266763)
Supplement: Attachment — Submitted filename: Comments- furazolidone associated fever.docx [file pone.0266763.s004.docx]

Congratulation to the corresponding author and team.

**Under the section of materials and method:**

It is mentioned that it was a retrospective case-control study, and it is also mentioned that the data was from July to September 2018. – When the study was conducted? When did the investigators called the subjects. 2019? / 2020?/ 2021? – This is to evaluate the reliability of the collected data on potential recall biases. Thank you.

Line 104 – did you exclude immunocompromised patients?

Line 113- Fever was defined as body temperature ≥ 38°C – How did the investigator know that the body temperature of the subjects (who reported as having drug fever) ≥ 38°C? Can you explain in methodology how the investigator gets the information when the nature of the study was retrospective and patients were treated as out-patient. The investigators called the subjects? How long a part? How you minimize recall biases? Who scanned the body temperature?

Line 115 – “ we conducted a follow up assessment…” – But this was a retrospective study… unless it was a retrospective, prospective 2 phases study. Appreciate if the author can clarify.

Line 122 – how the investigators obtained the WBC, Neutrophils and eosinophils? Did the subjects admitted to ward? Unless this was a prospective interventional study that specified the protocol to obtain blood investigations at specified date. Can the author please clarify?

The most important is the author able to convince the readers how reliable is the data? How comparable the control to case. Once this part is clarified the result and discussion they are fine to me.

Thank you very much!
