# Supplementary material for: Risk factors of furazolidone-associated fever
Source: PLoS One. 2022 Apr 8;17(4):e0266763. doi: 10.1371/journal.pone.0266763 (PMC8993017; doi:10.1371/journal.pone.0266763)
Supplement: Attachment — Submitted filename: Response to Reviewers.docx [file pone.0266763.s005.docx]

Thank you for your letter and for the reviewers' comments concerning our manuscript entitled “Risk Factors of Furazolidone-Associated Fever”. These comments are all valuable and very helpful for revising and improving our paper, as well as the important guiding significance to our research. We have studied comments carefully and have made corrections which we hope to meet with approval. The responds to the editor and reviewers' comments are as follows:

**Journal Requirements:**

Please review your reference list to ensure that it is complete and correct. If you have cited papers that have been retracted, please include the rationale for doing so in the manuscript text, or remove these references and replace them with relevant current references. Any changes to the reference list should be mentioned in the rebuttal letter that accompanies your revised manuscript. If you need to cite a retracted article, indicate the article’s retracted status in the References list and also include a citation and full reference for the retraction notice.

**Response:** Thank you for your suggestion. We have reviewed the format of references according to the authors' instructions of PLOS ONE and modified one by one according to it. We use the relevant current reference replace of the original reference 2, because the original reference is a Chinese literature, which cannot be found on the web of science or PubMed. The other reference we all have added the PMID numbers and made appropriate modifications to ensure that references are complete and correct.

**Reviewers' comments:**

**Reviewer's Responses to Questions**

**Comments to the Author**

1. If the authors have adequately addressed your comments raised in a previous round of review and you feel that this manuscript is now acceptable for publication, you may indicate that here to bypass the “Comments to the Author” section, enter your conflict of interest statement in the “Confidential to Editor” section, and submit your "Accept" recommendation.

Reviewer #1: All comments have been addressed

Reviewer #2: (No Response)

Reviewer #4: All comments have been addressed

________________________________________

2. Is the manuscript technically sound, and do the data support the conclusions?

The manuscript must describe a technically sound piece of scientific research with data that supports the conclusions. Experiments must have been conducted rigorously, with appropriate controls, replication, and sample sizes. The conclusions must be drawn appropriately based on the data presented.

Reviewer #1: Yes

Reviewer #2: Yes

Reviewer #4: Partly

**Response:** Yes, our manuscript described a technically sound piece of scientific research. The research has been conducted rigorously, with appropriate controls and sample sizes. This retrospective case-control study identified two risk factors for furazolidone-associated fever, which were female and clarithromycin. We also analyzed the characteristics of drug fever during anti-*Helicobacter pylori* therapy. These conclusions were drawn appropriately based on the data presented.

________________________________________

3. Has the statistical analysis been performed appropriately and rigorously?

Reviewer #1: Yes

Reviewer #2: Yes

Reviewer #4: Yes

________________________________________

4. Have the authors made all data underlying the findings in their manuscript fully available?

The PLOS Data policy requires authors to make all data underlying the findings described in their manuscript fully available without restriction, with rare exception (please refer to the Data Availability Statement in the manuscript PDF file). The data should be provided as part of the manuscript or its supporting information, or deposited to a public repository. For example, in addition to summary statistics, the data points behind means, medians and variance measures should be available. If there are restrictions on publicly sharing data—e.g. participant privacy or use of data from a third party—those must be specified.

Reviewer #1: Yes

Reviewer #2: (No Response)

Reviewer #4: Yes

**Response:** Yes, the data that support the findings of this study contain potentially sensitive patient information. Data are available from corresponding author Haibin Dai with the permission of the Ethics Committee of the 2^nd^ Affiliated Hospital, School of Medicine, Zhejiang University.

________________________________________

5. Is the manuscript presented in an intelligible fashion and written in standard English?

PLOS ONE does not copyedit accepted manuscripts, so the language in submitted articles must be clear, correct, and unambiguous. Any typographical or grammatical errors should be corrected at revision, so please note any specific errors here.

Reviewer #1: Yes

Reviewer #2: Yes

Reviewer #4: Yes

________________________________________

6. Review Comments to the Author

Please use the space provided to explain your answers to the questions above. You may also include additional comments for the author, including concerns about dual publication, research ethics, or publication ethics. (Please upload your review as an attachment if it exceeds 20,000 characters)

Reviewer #1: All the comments have been addressed

The data collection procedure is clear. The statistical analysis and data provided

to support the conclusion of the study are sufficient.

Reviewer #2: (No Response)

Reviewer #4: Congratulation to the author and team. It is an intersting findings particluarly in pharmacovigillence. Please refer to the attached comments.

Reviewer #4: 1) It is mentioned that it was a retrospective case-control study, and it is also mentioned that the data was from July to September 2018. – When the study was conducted? When did the investigators called the subjects. 2019? / 2020?/ 2021? – This is to evaluate the reliability of the collected data on potential recall biases. Thank you.

**Response:** Thank you for your suggestion. We feel sorry for the inconvenience brought to the you. The fact was that from July to September 2018, doctors in the fever clinic of our hospital reported many adverse reactions of drug fever caused by furazolidone (27 cases in total). When there are more than three cases of the same adverse reaction of the same drug per month, an investigation should be initiated in our hospital. I was one of the supervisors of adverse drug reactions in our hospital. The work of ADR supervisor was that we need to find out the causes of drug fever aggregation caused by furazolidone, track the outcomes of ADR and ask other patients used furazolidone whether they have febrile reactions. We changed use of furazolidone with different production batch numbers, checked the circulation and storage conditions of furazolidone, but found no possible causes of drug fever. After excluding drug quality, drug circulation, drug storage and other reasons, we considered looking for other risk factors of furazolidone-associated fever. We called the patients in 2018 and the study was also conducted in 2018. However, we are usually very busy with our work and not very proficient in English, this article has not be completed until 2021.

2) Line 104 – did you exclude immunocompromised patients?

Response: Thank you for your suggestion. We feel sorry that we only excluded the following patients (1) patients who were pregnant or breastfeeding; (2) patients who received furazolidone treatment not for Helicobacter pylori infection; (3) patients taking antibiotics or any acid suppressant or non-steroidal anti-inflammatory drug in the last 4 weeks; and (4) patients with chronic hepatic, renal, or pulmonary disease. Though immunocompromised patients did not meet the exclusion criteria of this study, we may have excluded partial of immunocompromised patients according to the present criteria.

3) Line 113- Fever was defined as body temperature ≥ 38°C – How did the investigator know that the body temperature of the subjects (who reported as having drug fever) ≥ 38°C? Can you explain in methodology how the investigator gets the information when the nature of the study was retrospective and patients were treated as out-patient. The investigators called the subjects? How long a part? How you minimize recall biases? Who scanned the body temperature?

**Response:** Thank you for your suggestion. I am one of the adverse drug reaction supervisors in our hospital. First of all, the doctors in the fever clinic reported adverse drug reaction of furazolidone-associated fever to us. Our hospital uses an in-hospital ADR reporting system, its interface as shown in the below. We got the general situation of adverse drug reactions from the hospital reporting system, and then got more information from medical records, such as body temperature. The 27 patients' body temperature was scanned by nurses. ADR supervisors telephoned the patients. One patient communicated by telephone for about 3 minutes. Three of the investigators in this study were ADR supervisors who were responsible for furazolidone-associated fever in 2018. ADR supervisors telephoned patients using furazolidone was documented on paper, which could avoid the recall biases of ADR supervisors. However, most patients received telephone follow-up one month after dispensing, and these patients may have some recall biases. The effects of recall bias we have mentioned in the limitations of this study, see lines 254-256. Thank you.

**The interface of hospital ADR reporting system**

4) Line 115 – “ we conducted a follow up assessment…” – But this was a retrospective study… unless it was a retrospective, prospective 2 phases study. Appreciate if the author can clarify.

**Response:** Thank you for your suggestion. We feel sorry for the inconvenience brought to the reviewer. We have shown in questions 1 and 3 that this study evolved during the investigation of the adverse reaction of furazolidone-associated fever. We did not attempt to analyze other risk factors until the end of the adverse reaction investigation when no other causes could be found. So, we think this was a retrospective study. Thank you.

5) Line 122 – how the investigators obtained the WBC, Neutrophils and eosinophils? Did the subjects admitted to ward? Unless this was a prospective interventional study that specified the protocol to obtain blood investigations at specified date. Can the author please clarify?

**Response:** Thank you for your suggestion. 27 Patients with fever who went back to hospital were assigned to the fever clinic for further consultation. Patients in the fever clinic will have routine blood tests. Routine blood tests were performed in all the 27 patients in the fever clinic. We collected their laboratory tests results from medical records. 27 patients with fever were only treated in the fever clinic and they did not admit to ward. Blood routine test was a routine examination in the fever clinic, not a clinical intervention in our study.

6) The most important is the author able to convince the readers how reliable is the data? How comparable the control to case. Once this part is clarified the result and discussion they are fine to me.

**Response:** We feel sorry for the inconvenience brought to the you. We have described the process of this study in detail in questions 1 to 5 at above. We really hope that we have clarified clearly to convince the readers. Thank you for your suggestion.

________________________________________

7. PLOS authors have the option to publish the peer review history of their article (what does this mean?). If published, this will include your full peer review and any attached files.

If you choose “no”, your identity will remain anonymous but your review may still be made public.

Do you want your identity to be public for this peer review? For information about this choice, including consent withdrawal, please see our Privacy Policy.

Reviewer #1: Yes: Sadia Shakeel

Reviewer #2: Yes: Dr. Gul Ambreen

Reviewer #4: No

Best wishes,

Yours sincerely,

Haibin Dai
